# Developing a Text Messaging Intervention to Prevent Binge and Heavy Drinking in a Military Population: Mixed Methods Development Study

**DOI:** 10.2196/55041

**Published:** 2024-03-19

**Authors:** Chase A Aycock, Indika Mallawaarachchi, Xin-Qun Wang, Daniel G Cassidy, Jordan M Ellis, Robert C Klesges, G Wayne Talcott, Kara Wiseman

**Affiliations:** 1 United States Air Force 37th Human Performance Squadron Joint Base San Antonio-Lackland Air Force Base San Antonio, TX United States; 2 Department of Public Health Sciences University of Virginia Charlottesville, VA United States; 3 United States Air Force 37th Training Wing Joint Base San Antonio-Lackland Air Force Base San Antonio, TX United States; 4 United States Air Force Wilford Hall Ambulatory Surgical Center, 59th Medical Wing Joint Base San Antonio-Lackland Air Force Base San Antonio, TX United States

**Keywords:** text messaging, alcohol reduction, binge drinking, US, United States, US military, alcohol misuse, military, functioning, readiness, health, career, careers, text message, text messages, short message service, SMS, SMS intervention, drinking, Air Force, Airmen, mixed methods approach, message, messages, development study, qualitative coding, drinking alcohol, alcohol consumption, survey, descriptive statistics

## Abstract

**Background:**

Alcohol misuse is the fourth leading cause of death in the United States and a significant problem in the US military. Brief alcohol interventions can reduce negative alcohol outcomes in civilian and military populations, but additional scalable interventions are needed to reduce binge and heavy drinking. SMS text messaging interventions could address this need, but to date, no programs exist for military populations.

**Objective:**

We aimed to develop an SMS text messaging intervention to address binge and heavy drinking among Airmen in Technical Training in the US Air Force.

**Methods:**

We implemented a 2-phase, mixed methods study to develop the SMS text messaging intervention. In phase 1, a total of 149 respondents provided feedback about the persuasiveness of 49 expert-developed messages, preferences regarding message frequency, timing and days to receive messages, and suggested messages, which were qualitatively coded. In phase 2, a total of 283 respondents provided feedback about the persuasiveness of 77 new messages, including those developed through the refinement of messages from phase 1, which were coded and assessed based on the Behavior Change Technique Taxonomy (BCTT). For both phases, mean persuasiveness scores (range 1-5) were calculated and compared according to age (aged <21 or ≥21 years) and gender. Top-ranking messages from phase 2 were considered for inclusion in the final message library.

**Results:**

In phase 1, top-rated message themes were about warnings about adverse outcomes (eg, impaired judgment and financial costs), recommendations to reduce drinking, and invoking values and goals. Through qualitative coding of suggested messages, we identified themes related to warnings about adverse outcomes, recommendations, prioritizing long-term goals, team and belonging, and invoking values and goals. Respondents preferred to receive 1 to 3 messages per week (124/137, 90.5%) and to be sent messages on Friday, Saturday, and Sunday (65/142, 45.8%). In phase 2, mean scores for messages in the final message library ranged from 3.31 (SD 1.29) to 4.21 (SD 0.90). Of the top 5 highest-rated messages, 4 were categorized into 2 behavior change techniques (BCTs): valued self-identity and information about health consequences. The final message library includes 28 BCTT-informed messages across 13 BCTs, with messages having similar scores across genders. More than one-fourth (8/28, 29%) of the final messages were informed by the suggested messages from phase 1. As Airmen aged <21 years face harsher disciplinary action for alcohol consumption, the program is tailored based on the US legal drinking age.

**Conclusions:**

This study involved members from the target population throughout 2 formative stages of intervention development to design a BCTT-informed SMS text messaging intervention to reduce binge and heavy drinking, which is now being tested in an efficacy trial. The results will determine the impact of the intervention on binge drinking and alcohol consumption in the US Air Force.

## Introduction

### Overview

Alcohol misuse contributes to 140,000 deaths per year, making it the fourth leading cause of death in the United States [1]. Alcohol use also contributes significantly to the epidemic of *deaths of despair*, both as a primary cause of death and as a contributor to approximately 25% of opiate overdose deaths and suicides [2,3]. Deaths from alcohol have increased by 37% since 2005 [4]. Binge drinking and the associated health and social consequences are substantial public health concerns, with a high prevalence among young adults [5,6].

Alcohol misuse is a significant problem in the US military because of its impact on military functioning and readiness and its effect on the health and careers of service members. The Millennium Cohort Study, sampling >77,000 troops (active duty and reserve/guard), indicated that 18.5% of respondents reported a history of alcohol problems, and 7% to 8% reported current weekly heavy drinking (ie, >14 and >7 drinks per week for men and women, respectively) [5]. In 2018, overall, 34% of all Department of Defense (DoD) service members reported binge drinking at least once a month, defined as ≥5 drinks per sitting for men and ≥4 drinks per sitting for women, with significant differences based on gender (35.2% and 28.2% for men and women, respectively, P<.05) [7]. Specifically, in the Air Force, 24.1% reported binge drinking in the past month. Overall, young adults drink more than any other age group, and on average, young adults in the military drink 22% more than demographically similar civilians (33.1% vs 27.1%) [5,8].

Fortunately, brief alcohol interventions (BAIs) have been validated in college students and community-dwelling young adults who report elevated drinking levels and alcohol-related health or social problems. Overall, BAIs (typically 1-2 individual counseling sessions that include personalized feedback on drinking that are delivered in a motivational interviewing style) result in 30% to 50% reduction in binge drinking, with positive treatment effects reported up to 4 years after the intervention [9-11].

While individually delivered BAIs are effective, they are challenging to implement in large populations, such as the US military, due to logistics and cost. Therefore, scalable and cost-effective approaches for reducing the prevalence of binge and heavy drinking in this population are needed. One very promising approach to cost-effectively disseminate BAI content to many thousands of at-risk service members is through the use of automated SMS text messages sent at key times during training or when participants may be at the greatest risk for binge drinking. For example, Cadigan et al [12] evaluated specific personalized feedback during football tailgating, an event heavily associated with binge drinking. College students (N=130) who reported a history of tailgating and binge drinking were randomized to 1 of 2 SMS text message groups: an event-specific SMS text message or generic alcohol education messages. At the 1-month follow-up, those in the event-specific SMS text message group reported lower estimated peak blood alcohol concentration than those who received generic alcohol education messages.

In another study, 765 young adults who screened positive for hazardous drinking were randomized to SMS text message assessments and feedback group, SMS text message assessments only group, or a no SMS text message control group over a 12-week period [13,14]. Overall, there were decreases in the number of self-reported binge drinking days from baseline in the SMS text message assessments and feedback group, whereas there were increases in binge drinking days in the SMS text message assessments only and no SMS text message groups. Overall weekend bingeing was also lowest in the SMS text message assessments and feedback group.

Finally, Teeters et al [15] tested a mobile phone–based intervention with personalized SMS text messages to determine its impact on driving after drinking. Participants included 84 college students who endorsed driving after drinking ≥3 drinks at least twice in the past 3 months. Participants were randomized to either information about driving after drinking (eg, how alcohol affected the brain and nervous system) or mobile phone–based BAI related to driving after drinking that included personalized feedback and interactive SMS text messaging. The mobile phone–based interventions included personalized feedback delivered via secure SMS text message (eg, normative feedback about drinking level and frequency of drinking and driving). The feedback was delivered in the context of a 15- to 20-minute, SMS text-based “session” that included open-ended, motivational interviewing–style SMS text messages asking participants about their reaction to the feedback, the potential personal and professional costs of being arrested for driving under the influence of alcohol (eg, losing their college scholarship), and their plans for avoiding drinking and driving in the future. The study clinicians made tailored empathic reflections about the participants’ reactions and encouraged specific goal setting/planning. The primary outcome variable was self-reports of driving after drinking at a 3-month follow-up. Results indicated that students receiving the mobile phone–based BAI reported significantly greater reductions in the likelihood of driving after drinking and the number of drinks consumed before driving than those in the information group.

While these findings suggest a potentially promising impact of SMS text message interventions on hazardous drinking outcomes, 2 recent meta-analyses in specific settings have shown potentially less robust efficacy of SMS text message interventions on heavy drinking when delivered as the only intervention [16] and when delivered as a preventive intervention [17]. For example, when delivered as the only intervention, Bendtsen et al [16] determined that while heavy episodic drinking and weekly alcohol consumption were low among participants who received the SMS text message intervention, the CI of these estimates both included the null, indicating a lack of a statistically significant association. In addition, the included studies were judged to be of low quality [16]. Therefore, more studies are needed to fully understand the potential impact of SMS text message interventions on binge and heavy drinking.

To date, the few studies that evaluated SMS text messages for binge drinking have been conducted in civilian settings. However, a large percentage of military personnel are also similar in age to college populations, making them potentially similarly receptive to mobile health interventions. Military personnel may also have confidentiality and health stigma concerns, which mobile health interventions are well suited to mitigate. Furthermore, military populations have expressed general interest in digital interventions [18,19]. As mentioned previously, the military has a particular problem with binge and heavy drinking. In all service branches, there is a protracted ban of alcohol in Basic Military Training and the first part of Technical (advanced) Training. In the Air Force, the ban is for approximately 12 weeks and tolerance to alcohol, even if the Airman (note that *Airman* is the term used by the Air Force for its service members, regardless of gender or position in the Air Force) used to drink alcohol before basic training, tolerance is low at the completion of training. Our research team has successfully implemented a group-based BAI and observed a significant reduction in the odds of an alcohol-related incident (ARI; getting in trouble while drinking) over the year of the intervention compared to the previous year (odds ratio 0.56, 95% CI 0.38-0.81; P=.002) [20]. Implementing an SMS text message intervention to complement this group-based BAI when Airmen begin to be allowed to leave base and access alcohol for the first time in approximately 3 months has enormous potential.

As the types of messages that civilians find persuasive may not be persuasive to military personnel, it is crucial to develop messages using feedback directly from Airmen in Technical Training to find messages that will be compelling and influential for this population. While individually tailoring the messages is often presumed to be beneficial in targeting addictive behaviors, there are also benefits to having universally acceptable messages. Nontailored messages require no information about the recipient, potentially diminishing the service members’ concerns regarding anonymity. In addition, nontailored messaging can increase feasibility and future dissemination if the intervention is determined to be effective.

### Objectives

This paper describes a 2-phase mixed methods study designed to develop an SMS text message intervention to reduce binge and heavy drinking among Airmen in Technical Training. Specifically, the aims of this study were to (1) determine the persuasiveness of SMS text messages suggested by experts and Airmen designed to discourage binge and heavy drinking, (2) obtain data about the dose and timing of messages, and (3) create a final message library for efficacy testing.

## Methods

### Phase 1

#### Study Population

Participants included 149 Airmen in Technical Training located across 4 US Air Force bases who were attending, as part of standard training, a squadron-level group BAI session. Surveys were administered in each squadron at the end of the BAI session.

#### Procedures

##### Message Development

An initial set of 49 messages were created by content and population experts (eg, military, retired military, and nonmilitary psychologists and health behavior change experts). To reduce participant burden, the 49 messages were divided randomly into 3 sets, with 16 to 17 messages in each set.

##### Message Evaluation

All Airmen were asked to provide their age, gender, and past-month binge drinking frequency (“During the past 30 days, how many times did you have 4 (for women)/5 (for men) or more alcoholic drinks on one occasion?” with response options, “never,” “1 or 2 times,” “3 to 5 times,” “6 to 10 times,” and “11 or more times”). Overall, 3 questions assessed general beliefs about the importance of being successful in Technical Training, perceived impact of receiving a punishment related to alcohol while in the Air Force, and level of concern about having problem drinking, all of which used a 5-point Likert-type scale ranging from 1 (strongly disagree) to 5 (strongly agree). The Airmen were then presented with their set of messages to be evaluated. All 3 sets of messages were included equally in each survey administration, so that all 49 items were assessed evenly within each squadron. For each message, the Airmen responded to 2 items (ie, “This statement makes me concerned about the impact of drinking while in Technical Training” and “This statement discourages me from wanting to drink alcohol excessively”) using a 5-point Likert-type scale ranging from 1 (strongly disagree) to 5 (strongly agree) [21]. Following message rating, the Airmen were asked, “What might be a good text message that you can think of that might discourage an Airmen from drinking too much during Technical Training?” and provided space for a write-in response. Finally, the Airmen were asked about message timing and frequency and for specific points during their training when information of this nature might be particularly useful to receive.

##### Data Analysis

Following data entry, all responses were summarized using descriptive statistics. The Airmen’s age was dichotomized to <21 years or ≥21 years to reflect US legal drinking limits. Airmen who reported any binge drinking in the past month were coded as binge drinkers. To explore the messages’ persuasiveness, means, medians, and SDs were calculated for the persuasiveness item, “This statement discourages me from wanting to drink alcohol excessively” for each message. The messages were ranked based on persuasiveness and compared according to age and gender using Wilcoxon rank sum tests to identify the top-ranked messages with no statistically significant differences in rating based on age or gender. The resulting top messages were qualitatively coded for themes by 3 study authors (CAA, DGC, and JME). The frequency and timing of message delivery was explored using frequencies and percentages. Finally, messages suggested by Airmen were qualitatively coded for themes.

### Phase 2

#### Study Population

Participants included 283 Airmen in Technical Training located across 4 US Air Force bases who were attending, as part of standard training, a squadron-level group BAI session. Surveys were administered in each squadron at the end of the BAI session.

#### Procedures

##### Message Development

Messages suggested by Airmen from phase 1 were refined, often by reformatting them into Airmen quotes, and coded based on the Behavior Change Technique Taxonomy (BCTT). Highly rated messages from phase 1 were also refined and coded based on the BCTT. Informed by the study by Michie et al [22], additional expert-created messages were developed to ensure a pool of messages that addressed key behavior change techniques (BCTs) previously identified to successfully reduce alcohol consumption. The final pool of 77 messages covered the following BCTs: problem solving, goal setting (outcome), action planning, discrepancy between current behavior and goal, review outcome goals, self-monitoring of behavior, information about consequences, information about social and environmental consequences, anticipated regret, social comparison, behavior substitution, comparative imagining of future outcomes, future punishment, reducing negative emotions, restructuring the social environment, avoiding/reducing exposure to cues for the behavior, identification of self as a role model, valued self-identity, and verbal persuasion about capability. To reduce participant burden, the 77 messages were divided randomly into 5 sets, grouped according to BCTs, with 15 to 16 messages in each set.

##### Message Evaluation

All Airmen were asked to provide their age, gender, and past-month binge drinking frequency (“During the past 30 days, how many times did you have 4 (for women)/5 (for men) or more alcoholic drinks on one occasion?” with the following response options: “never,” “1 or 2 times,” “3 to 5 times,” “6 to 10 times,” and “11 or more times”). Overall, 8 questions were included to assess general beliefs about the importance of being successful in Technical Training, perceived impact of receiving a punishment related to alcohol while in the Air Force, level of concern about having problem drinking, perceived culture of drinking in the Air Force, and opinions about alcohol reduction strategies and not drinking while in Technical Training, all of which used a 5-point Likert-type response scale ranging from 1 (strongly disagree) to 5 (strongly agree). The Airmen were then presented with their set of messages to be evaluated. All 5 sets of messages were included equally in each survey administration, so that all 77 items were assessed evenly within each squadron. For each message, the Airmen responded to 4 items to assess persuasiveness (ie, “This statement makes me concerned about the impact of drinking while in Technical Training”; “This message makes me think about how to be successful in Technical Training”; “This statement discourages me from wanting to drink alcohol excessively”; and “This message would be useful if I wanted to reduce or limit my drinking”) using a 5-point Likert-type scale ranging from 1 (strongly disagree) to 5 (strongly agree) [21]. Following message rating, the Airmen were asked to select 3 messages from their set that they believed would be most likely and least likely to help Airmen make good choices about alcohol.

##### Data Analysis

Following data entry, all responses were summarized using descriptive statistics. The Airmen’s age was dichotomized to <21 years or ≥ 21 years to reflect US legal drinking limits. Airmen who reported any binge drinking in the past month were coded as binge drinkers. To determine message suitability for inclusion in the final message pool, means, medians, and SDs were calculated for each item regarding message persuasiveness. The messages’ persuasiveness was compared according to age and gender using Wilcoxon rank sum tests to identify any messages with statistically significant differences in rating based on age or gender. The messages in phase 2 were ranked based on persuasiveness and considered within each BCT and overall to develop a final message pool that could address multiple BCT targets. Any messages that were statistically different based on age or gender where 1 group had a mean score below a threshold of 3.5 were excluded from consideration.

### Ethical Considerations

In both phases, participants were asked to complete an anonymous survey. Both surveys were approved by the Joint Base San Antonio institutional review board as quality improvement, and thus, a full review was not required. The voluntary, fully informed consent of participants was obtained as required by 32 Code of Federal Regulations (CFR) 219 and Department of Defense Instruction (DODI) 3216.02. The study staff emphasized that participation in the survey was optional and anonymous, and >99% of the population consented to participate. No information was obtained about the individuals who did not choose to participate.

## Results

### Phase 1

Among the 149 respondents, 113 were men, 76 were aged <21 years, and 131 reported no heavy drinking in the 30 days before starting military training (Table 1). Approximately all (140/148, 94.6%) reported agreement with the statement, “Being successful in Technical Training is extremely important to me.” Motivation to be successful in Technical Training was also high; 89.9% (133/148) agreed to the statement, “Receiving punishment for an alcohol related incident (ARI) in Technical Training could harm or end my career,” and 91.2% (135/148) agreed that “Whether I drink alcohol or not, it is very important to me to NOT have a problem related to my drinking” (data not shown).

**Table 1:** Descriptive characteristics of the study populations in phases 1 and 2^a^.

Characteristics		Phase 1 (n=149), n (%)		Phase 2 (n=283), n (%)	
**Gender**					
	Woman		33 (22.6)		90 (32.3)
	Man		113 (77.4)		189 (67.7)
**Age (y)**					
	<21		76 (51.4)		125 (46.3)
	≥21		72 (48.6)		145 (53.7)
**Binge drinking in the past 30 days**					
	Any		17 (11.5)		73 (26.4)
	None		131 (88.5)		204 (73.6)

^a^The values do not add up to 100% owing to missing data.

The 10 expert-created messages with highest median persuasiveness scores and statistically similar responses based on age (<21 years and ≥21 years) and gender (man and woman) are shown in Table 2. Qualitative coding indicated that 9 of the 10 (90%) top-rated messages had a theme related to warning about adverse outcomes, with specific adverse events related to the following: impaired judgment (4/9, 44%), career or receiving an ARI (3/9, 22%), assault (1/9, 11%), and financial costs (1/9, 11%). Of the 10 top-rated messages, 5 (50%) also included recommendations, with specific recommendations related to the following: planning (3/5, 60%), moderation (1/5, 20%), and low-risk drinking (1/5, 20%). Of the 10 top-rated messages, 1 (10%) invoked values and goal reminders.

**Table 2:** Top-rated, expert-created messages among respondents, with theme coding performed by the study team in phase 1^a,b,c^.

Themes and expert-created messages				Score, mean (SD)	
**Warning about adverse outcomes**					
	**Career**				
		“You should develop a plan if you drink alcohol to avoid the risk of harming your Air Force career.”			4.21 (0.90)
		“No one who gets an ARI^d^ ever planned to get one.”			3.92 (1.27)
		“An ARI is potentially career ending in the Air Force.”			3.78 (1.34)
	**Impaired judgment**				
		“Drinking can lower your inhibitions, leading to poor social judgement. Limit your drinking or don’t drink at all.”			4.13 (1.08)
		“Drinking impairs your judgement. Plan to keep your drinking under control so you won’t make decisions that you later regret.”			4.13 (1.02)
		“Set your limit ahead of time. Once you’ve had two drinks, you’re no longer the best judge of what should come next.”			4.02 (1.13)
		“Drinking can lead to aggression (verbal and physical). If you are going to drink, avoid people and places that could lead to conflicts.”			3.96 (0.98)
	**Assault**				
		“WARNING, almost all sexual assaults in Technical Training involved heavy drinking.”			4.06 (0.88)
	**Financial costs**				
		“You’ve earned that paycheck, so make it last. Consider slowly sipping on a drink instead of gulping it. Non-alcoholic drinks cost much less than alcoholic ones. Water is free.”			4.00 (1.07)
**Recommendation**					
	**Planning**				
		“You should develop a plan if you drink alcohol to avoid the risk of harming your Air Force career.”			4.21 (0.90)
		“You want to have a successful plan for your future in the Air Force and are willing to take the time to work at making responsible decisions when it comes to drinking alcohol.”			4.10 (0.95)
		“Set your limit ahead of time. Once you’ve had two drinks, you’re no longer the best judge of what should come next.”			4.02 (1.13)
	**Moderation**				
		“Drinking can lower your inhibitions, leading to poor social judgement. Limit your drinking or don’t drink at all.”			4.13 (1.08)
	**Low-risk drinking**				
		“Drinking impairs your judgement. Plan to keep your drinking under control so you won’t make decisions that you later regret.”			4.13 (1.02)
**Reminder of values and goals**					
	“You want to have a successful plan for your future in the Air Force and are willing to take the time to work at making responsible decisions when it comes to drinking alcohol.”		4.10 (0.95)		

^a^Mean scores for the item, “This statement discourages me from wanting to drink alcohol excessively.”

^b^Response range 1-5 [21].

^c^Messages could have multiple theme codings and appear more than once in the table.

^d^ARI: alcohol-related incident.

Exemplar messages suggested by Airmen are shown in Table 3. Qualitative coding indicated that among the 13 exemplar messages, 11 (85%) had a theme or themes related to warning about adverse outcomes, with specific adverse events related to career (6/11, 55%) and impaired judgment (1/11, 9%) or nonspecified consequences (5/11, 45%). Of the 13 exemplar messages, 5 (39%) included recommendations, all with specific recommendations related to prioritizing long-term goals. In addition, of the 13 messages, 2 (15%) contained themes related to team and belonging and 2 (15%) contained themes related to values and goal reminders.

**Table 3:** Messages suggested by Airmen respondents to discourage peers from excessive drinking during Technical Training, with theme coding performed by the study team in phase 1.

Messages suggested by Airmen	Theme coding
“Alcohol lasts a night; paperwork goes on your record. Don’t risk it.”	Warning about adverse outcomes (career) Recommendation (prioritize long-term goals)
“Choose carefully if you want to ruin your life just for one night of fun.”	Warning about adverse outcomes (nonspecific) Recommendation (prioritize long-term goals)
“Do not let actions taken while drunk ruin how far you’ve come and ruin the rest of your life. Think, ‘Is this what I would do if I were sober?’”	Warning about adverse outcomes (nonspecific) Warning about adverse outcomes (impaired judgment) Recommendation (prioritize long-term goals)
“Don’t throw away your money and career this early on alcohol just to have some ‘fun’.”	Warning about adverse outcomes (career) Recommendation (prioritize long-term goals)
“Enjoy yourself off base; however, just remember that your actions have consequences, which includes drinking.”	Warning about adverse outcomes (nonspecific)
“First the man takes the drink, then the drink takes the man.”	Warning about adverse outcomes (nonspecific)
“Live in the moment but make sure that moment doesn’t destroy your future.”	Warning about adverse outcomes (nonspecific)
“If you drink, we won’t be on the same team anymore.”	Warning about adverse outcomes (career) Team and belonging
“Remember what the Air Force represents.”	Reminder about values and goals
“Remember, having casual drinks with friends is fine, but know your limit and be responsible. Aim High Airman!”	Warning about adverse outcomes (career) Values and goals reminder
“We have made so many memories together and have many more to go on. Make good choices.”	Team and belonging
“Why risk a lifelong career for a few drinks?”	Warning about adverse outcomes (career) Recommendation (prioritize long-term goals)
“Your career could be in that bottle.”	Warning about adverse outcomes (career)

Most Airmen preferred to be sent 1 to 3 messages per week (124/137, 90.5%), followed by 4 to 6 messages per week (10/137, 7.3%). The most frequently selected days to receive messages were Friday, Saturday, and Sunday in combination (65/142, 45.8%) and Thursday, Friday, and Saturday in combination (20/142, 14.1%). Most respondents preferred messages to be sent in the early evening (84/149, 56.4%) or late evening (80/149, 53.7%; data not shown).

### Phase 2

Among the 283 respondents, 189 were men, 125 were aged <21 years, and 204 reported no heavy drinking in the 30 days before starting military training (Table 1). Approximately all (243/259, 93.8%) reported agreement with the statement, “Being successful in Technical Training is extremely important to me.” Motivation to be successful in Technical Training was also high, as 90.3% (233/258) agreed to the statement, “Receiving punishment for an alcohol related incident (ARI) in Technical Training could harm or end my career,” and 88.7% (228/257) agreed that “Whether I drink alcohol or not, it is very important to me to NOT have a problem related to my drinking” (data not shown).

The final messages selected for inclusion in the SMS text message intervention are shown in Table 4. The final message library includes 28 BCTT-informed messages across 13 different BCTs and 5 messages (not shown) directly referencing content originally introduced in the group BAI. The program is designed to be delivered over 6 weeks, sending 3 to 4 messages per week, and most messages are sent on Friday and Saturday in the late afternoon and early evening. Mean scores for the included messages ranged from 3.31 (SD 1.29) to 4.21 (SD 0.90). Of the top 5 highest-rated messages in the final message library, 4 were categorized into 2 BCTs: valued self-identity and information about health consequences. Specific recommendations for behavior differ for those who are and those who are not legally allowed to drink in the United States, as those with ARIs and aged <21 years face harsher disciplinary action. Therefore, while designed to be a universally delivered program, for Airmen aged 21 years or those who will be aged 21 years during the intervention period, safe drinking–related messages were prioritized, whereas for Airmen aged <21 years, abstinence-related messages were prioritized. The total number of messages received by each Airmen is the same. Therefore, the distribution of BCTs differs according to age, with most action planning messages (3/4, 75%) only being sent to Airmen aged ≥21 years. Messages for Airmen aged <21 years have a high percentage of messages addressing the consequences of alcohol use. The inclusion of targeted messages based on age also resulted in 2 new messages needing to be created that were informed by phase 2 results but not evaluated by Airmen. Of the final 28 messages, 8 (28.6%) were informed by messages suggested by Airmen from phase 1.

**Table 4:** The final message library of an SMS text message intervention to prevent binge and heavy drinking among Airmen in Technical Training, with coding based on Behavior Change Technique Taxonomy.

Themes and messages			Score, mean (SD)
**Problem solving**			
	“Write down your money and career goals. How does alcohol fit into the picture? How would a DUI^a^ or an ARI^b^ impact those goals?”	4.07 (1.19)	
**Goal setting (outcome)**			
	“Only you can choose how to spend your free time. What are options that are most in line with your long-term goals and plans?”	3.82 (1.22)	
**Action planning**			
	“If you drink, eat a meal beforehand and consider having a glass of water between drinks.”^c^	3.69 (1.21)	
	“Before going out, think about choosing a Wingman who will not drink at all and who will be a designated driver.”^c^	3.62 (1.29)	
	“If you drink tonight, don’t drive. Call an Uber [link to Uber] or a Lyft: [link to Lyft]”^c^	3.57 (1.15)	
	“You can help prevent an ARI for you or a friend by helping them get home safely. Call them an Uber [link to Uber] or a Lyft [link to Lyft]”^d^	—^e^	
	“Your tolerance to alcohol will probably be very low after not drinking for several weeks. If you are going to drink, consider limiting your alcohol to three or less drinks.”^c^	3.58 (1.26)	
**Review outcome goals**			
	“Advice from a fellow Airman: ‘Think about what truly matters to you in the long run.’”^f^	3.90 (1.19)	
**Self-monitoring of behavior**			
	“If you choose to drink, partner with a Wingman to keep your drink count to less than 3 per outing and 1 per hour.”^c^	3.43 (1.25)	
**Information about consequences**			
	“Imagine failing Technical Training because of a drinking incident. Is it worth the risk? What’s your plan to make sure alcohol use doesn’t derail your career goals?”^d,f^	4.16 (1.08)	
	“Alcohol lasts a night, but paperwork stays forever on your record. What can you do to make sure today’s decisions do not hurt your tomorrows?”^d,f^	4.07 (1.08)	
	“Underage drinking or legal drinking that results in an adverse incident can both lead to an ARI. What would be the impact of an ARI on your long-term goals?”^d^	3.96 (1.08)	
	“Advice from a fellow Airman: ‘Live in the moment but make sure that moment doesn’t hurt your future.’”^f^	4.00 (0.99)	
	“Choosing to not drink can lead to better sleep and feeling refreshed and rested each morning. What would you like to get out of each day?”	3.85 (1.17)	
	“We are all in this together for the good times and the bad. Remember, if one of us gets in trouble, the whole squadron will have to suffer the consequences.”^f^	3.87 (1.37)	
**Social comparison**			
	“Many of your fellow Airmen choose not to drink at all during Tech Training to avoid getting side-tracked. What will you choose to do?”	3.71 (1.23)	
	“46% of Airmen choose not to drink in technical school. Consider calling a friend and seeing if they’d like to do something alcohol free this weekend.”	3.31 (1.29)	
**Behavior substitution**			
	“One of the best ways to prevent hazardous drinking is to participate in fun alcohol-free group activities. What plans can you make this weekend to keep everyone entertained, safe, and out of trouble?”^d^	3.83 (1.06)	
	“Interested in an alcohol-free weekend? Click here [Link to study webpage listing alcohol-free activities by base] to see the top things people do to have fun around here.”	3.81 (1.25)	
**Comparative imagining of future outcomes**			
	“Take a moment to reflect on what you hope to achieve in the next year, academically, professionally, or athletically. How could alcohol get in the way?”	4.19 (0.95)	
**Future punishment**			
	“Underage drinking is a primary reason Airmen get Article 15s and those can lead to a separation. What are other ways you can have fun?”^d,f^	3.65 (1.16)	
	“Drinking can be a reason why Airmen get Article 15s and those can lead to a separation. What are other ways you can have fun?”^c^	—	
**Reduce negative emotions**			
	“Alcohol is not the only way to unwind. Many Airmen enjoy doing things that do not include alcohol. Local alcohol-free activities include movies, coffee/desert shops, trampoline parks, playing video games or sports, or visiting a museum. What might you like to do that is alcohol-free?”	3.98 (1.13)	
	“There are healthier, safer, and cheaper ways to manage stress than drinking. Consider going to the gym or calling a friend.”	3.94 (1.02)	
	“Tech Training is challenging, and it’s helpful to have ways to relax and unwind. Have you ever tried tactical breathing? Find out more: [link to breathing exercise website].”	3.86 (0.97)	
**Identification of self as a role model**			
	“Evenings out are a good time to be a responsible Wingman. Set the example and others will follow.”^f^	3.91 (1.21)	
**Valued self-identity**			
	“Remember why you joined the Air Force. [link to inspirational video]. Don’t let alcohol be what stops you from achieving these goals.”^f^	4.21 (1.08)	
	“You are among the 1% who chose a profession of arms. Consider the role you would like alcohol to have within your professional identity.”	4.00 (1.17)	

^a^DUI: driving under the influence of alcohol.

^b^ARI: alcohol-related incident.

^c^Only sent to Airmen who are of US legal drinking age or who will be before the end of the intervention period.

^d^Only sent to Airmen who are younger than the US legal drinking age.

^e^Indicates a message that was not included in phase 2 testing.

^f^Original source was a message suggested by Airmen from phase 1.

## Discussion

### Principal Findings

This 2-phase study developed an automated SMS text message intervention designed to reduce binge and heavy alcohol use among young adults entering the military. In phase 1, a sample of US Air Force Technical School trainees rated the messages created by experts and provided suggestions. In phase 2, the messages suggested by Airmen and those that were highly rated in phase 1 were refined and rated alongside additional expert-created messages. These phases culminated in the creation of a BCTT-informed SMS text message program for Airmen in Air Force Technical Training and highlight an approach for message testing and program development, which incorporates mixed methods feedback from the target population. This approach allowed us to have a final program that was directly informed by the target population and led to more than one-fourth (8/28, 28.6%) of the final messages being in the Airmen’s own words.

Top-rated, expert-created messages from phase 1 included recommendations for planning or drinking in moderation and warnings about adverse outcomes associated with drinking in excess (eg, career impact, risk for sexual assault, and impaired judgment associated with drinking). In particular, most (9/10, 90%) of the top-rated messages in phase 1 were loss-framed messages. Messages suggested by both experts and Airmen included warnings about adverse outcomes, especially pertaining to one’s career. Other themes of the messages suggested by Airmen were reminders to prioritize long-term goals and values over short-term “fun” and emphasis on belonging to the same collective team. In phase 2, an expanded set of items organized based on BCTs allowed for a more nuanced analysis of the potential message pool. Specifically, it also allowed for the inclusion of more gain-framed messages. As in phase 1, the highly rated messages in phase 2 were related to themes such as adverse outcomes and long-term values and goals. The 2 highest-rated messages in the final program were related to the topics of valued self-identity (“Remember why you joined the Air Force. [link to inspirational video]. Don’t let alcohol be what stops you from achieving these goals.”) and comparative imagining of future outcomes (“Take a moment to reflect on what you hope to achieve in the next year, academically, professionally, or athletically. How could alcohol get in the way?”), which both had loss-framed messages around alcohol *getting in the way*. Thematic consistency of top-rated messages across both phases of the study suggests that the messages ultimately included in the final message pool are likely to resonate with the target population. The real-world effectiveness of these messages are being empirically evaluated in an ongoing clinical trial.

The final intervention is targeted at Airmen of the US legal drinking age, while also being designed to be uniformly acceptable across genders. Therefore, message ratings were considered overall and according to age and gender. This allowed for a program that can still by scaled up easily while providing relevant content for specific subsets of the population. If tailoring based on other factors becomes necessary, for example, the Airman’s specific role in the Air Force, this study provides a framework through which messages can be developed and evaluated with specific tailoring needs in mind. It will also be important to explore the potential moderators of program success after completion of the currently underway clinical trial. The presence of a strong effect modifier would suggest a potential need to tailor based on specific Airmen characteristics and would help justify the collection of additional personal information at the time of program sign-up.

Phase 1 also identified Airmen’s preferences for receiving SMS text messages, which were incorporated into the final intervention. Specifically, the Airmen preferred to receive messages at a frequency of approximately 3 to 5 times per week, in the evenings and on weekends. This could be an ideal window to receive a timely prompt in the form of an SMS text message, given that Airmen are most at risk for binge drinking from Friday evening to Sunday evening when they become able to leave their base (approximately 10-12 weeks after starting Basic Military Training). Having information about message timing and frequency preferences allowed us to develop an SMS text message program that should fit within each Airman’s daily routine and maximizes the number of messages sent at times when the Airmen may be considering and planning off-base activities, where alcohol is more readily available.

Across both phases, respondents reported interest in being successful in Technical Training, believed that an ARI would be detrimental to their careers, and did not want to have a problem associated with their drinking. These results should be interpreted with some caution, as respondents had just completed the group BAI; therefore, the Airmen might have been having heightened feelings regarding the potential harms of alcohol use. It could also show the potential of the group BAI to be a teachable moment, which the SMS text message intervention will attempt to capitalize on to sustain self-reflection and opinions about alcohol use and the desire for success during Technical Training. The sentiment of success during Technical Training is also referenced in the SMS text message intervention to increase the potential salience of the messages.

In addition to being developed specifically for a noncivilian population, there are other differences between the SMS text message intervention described in this paper and the previously developed alcohol reduction SMS text message interventions [12,13]. For example, the 2 previous studies designed to address binge drinking included assessments of drinking behavior. Specifically, Cadigan et al [12] collected information about participant drinking behavior while tailgating at baseline to provide normative feedback to participants during the intervention, which referenced back to the information each participant provided. Suffoletto et al [13] asked participants to report anticipated drinking behavior and the highest number of drinks consumed over the weekend, and responses were used to tailor other intervention messages. Owing to confidentiality concerns within the military, actual alcohol use behavior is not able to be captured by Airmen within the SMS text message intervention described in this paper. Thus, direct normative feedback cannot be delivered to participants. However, there is a message included to allow for social comparison. Despite this difference, there are also similarities between the proposed intervention and previous studies, such as sending messages when participants are at the highest risk of drinking (ie, weekends) and not tailoring the messages based on other personal characteristics.

In addition to the programmatic differences described previously, to the best of our knowledge, the SMS text message intervention described in this paper is the first to use BCTs to guide intervention development within the context of alcohol reduction [22,23]. The use of BCTs is helpful for understanding the hypothesized mechanisms of action through which the SMS text message intervention may reduce binge and heavy alcohol use. In addition, it allows for the testing of active components in future evaluations. This SMS text message intervention includes content that covers 13 distinct BCTs, which is more than the average number seen in previous behavior change interventions to reduce alcohol use (mean 9.1 BCTs) [24], but less than another SMS text message intervention that was coded based on BCT after its development and evaluation (23 BCTs) [23]. However, there may be other BCTs that could prove to be valuable. A future study could evaluate the impact of adding content that addresses additional BCTs. Moreover, the SMS text message intervention could be separated into unique BCT components and compared directly [25] to better understand which BCTs are most important for addressing binge and heavy drinking in this population.

### Limitations

A limitation of this study is that this pool of messages is based solely on the Airmen’s predictions about which messages will influence future drinking behavior. Consequently, the final message pool must be empirically tested to determine its impact on alcohol-related behavior. This study is the first to develop an SMS text message intervention to reduce binge and heavy drinking within a US military population, specifically, the US Air Force. However, it is possible that the results of this mixed methods approach are not generalizable to other military settings.

### Conclusions

This study included the involvement of members from the target population throughout the formative stages of intervention development, to design an SMS text message intervention to reduce binge and heavy drinking in the US Air Force. Message content was anchored to evidence-based behavior change techniques according to the BCTT in conjunction with integration of feedback from Airmen regarding SMS text messaging language, timing, and frequency, thus increasing the odds of a robust intervention effect.

## Abbreviations

ARI: alcohol-related incident
BAI: brief alcohol intervention
BCT: behavior change technique
BCTT: Behavior Change Technique Taxonomy
CFR: Code of Federal Regulations
DoD: Department of Defense
DoDI: Department of Defense Instruction

## Data Availability

The data sets generated and analyzed during this study are available from the corresponding author upon reasonable request.
